# Insulin and glucose responses to hypoxia in male and female neonatal rats: Effects of the androgen receptor antagonist flutamide

**DOI:** 10.14814/phy2.14663

**Published:** 2021-01-04

**Authors:** Santiago Rolon, Christine Huynh, Maya Guenther, Minhal Gardezi, Jonathan Phillips, Ashley L. Gehrand, Hershel Raff

**Affiliations:** ^1^ Endocrine Research Laboratory Aurora St. Luke's Medical Center Advocate Aurora Research Institute Milwaukee WI USA; ^2^ Department of Medicine Medical College of Wisconsin Milwaukee WI USA; ^3^ Department of Surgery Medical College of Wisconsin Milwaukee WI USA; ^4^ Department of Physiology Medical College of Wisconsin Milwaukee WI USA

**Keywords:** HOMA‐IR, insulin resistance, newborn, oxygen, sexual dimorphism

## Abstract

Hypoxia is common with preterm birth and may lead to long‐term effects on adult pancreatic endocrine function and insulin sensitivity. This phenomenon may be sexually dimorphic due to the hypoxia‐induced augmentation of the neonatal androgen surge in male newborns. We evaluated this phenomenon by pretreating neonatal rats on postnatal days (PD) 1, 6, 13, or 20 with flutamide (a nonsteroidal androgen receptor antagonist) at a standard or a high dose (10 or 50 mg/kg) compared to vehicle control. One day later, neonatal rats were exposed to either acute normoxic or hypoxic separation (fasting) for 90 min, and blood was sampled for the measurement of insulin and glucose and the calculation of HOMA‐IR as an index of insulin resistance. During normoxic and hypoxic separation (fasting), flutamide increased insulin secretion in PD2, PD7, and PD14 pups, high dose flutamide attenuated insulin secretion, and high dose flutamide attenuated the increase in HOMA‐IR due to hypoxia. Our studies suggest a unique role of the androgen receptor in the control of neonatal pancreatic function, possibly by blocking a direct effect of neonatal testosterone or in response to indirect regulatory effects of androgens on insulin sensitivity.

## Graphical abstract

Hypoxia is common with preterm birth and may lead to long‐term effects on adult pancreatic endocrine function and insulin sensitivity. This phenomenon may be sexually dimorphic due to the hypoxia‐induced augmentation of the neonatal androgen surge in male newborns. Our studies suggest a unique role of the androgen receptor in the control of neonatal pancreatic function, possibly by blocking a direct effect of neonatal testosterone or in response to indirect regulatory effects of androgens on insulin sensitivity.

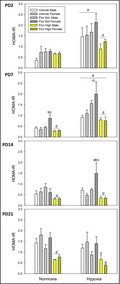

## 1 INTRODUCTION

Hypoxia is a common stressor in premature neonates that may have long‐term effects on adult endocrine function, including insulin and glucose dynamics as well as control of the hypothalamic–pituitary–adrenal (HPA) axis which is involved in the control of the sensitivity to insulin in the target tissue (Aynsley‐Green et al., 1997; Chan et al., 2001; Chintamaneni et al., 2013; Davis et al., 1999; Guenther et al., 2012; Iiyori et al., 2007). We have previously shown the short‐term effects of androgen regulation on the HPA axis in the neonatal rat (Rolon et al., 2019). It seems that androgen secretion may also play a key role directly in the control of rat pancreatic beta‐cell function as well as the overall regulation of plasma insulin and glucose via effects on insulin sensitivity (Harada et al., 2018, 2019; Li et al., 2008; More, Mishra, Gopalakrishnan, et al., 2016; Pignatelli et al., 2006; Zhou et al., 2017). This is a follow‐up study of a cohort of neonatal rats treated with flutamide (Eulexin®), an androgen receptor (AR) antagonist, and subsequently exposed to acute normoxic or hypoxic separation (with acute fasting). Flutamide is a nonsteroidal androgen receptor (AR) antagonist used clinically in adults. This study addresses the effects of the androgen receptor and its blockade on plasma insulin and glucose as well as an index of insulin resistance.

It is well known that subsequent sexually dimorphic metabolic phenotypes may result from the neonatal testosterone surge in male rat pups the effect of which is mediated by the androgen receptor (Gehrand et al., 2016). However, subsequent metabolic effects of neonatal androgens in female rats could result from the production of adrenal androgen precursors (Pignatelli et al., 2006; Zhou et al., 2015). While the adult rat adrenal does not express CYP17 and cannot synthesize appreciable androgens (Gallo‐Payet & Battista, 2014), the neonatal rat adrenal may have the capacity to synthesize androgens that function as critical regulators of normal development and could possibly account for non‐sexually dimorphic effects of androgen receptor actions (Harada et al., 2018, 2019; Li et al., 2008; More, Mishra, Gopalakrishnan, et al., 2016; More, Mishra, Hankins, et al., 2016; Pignatelli et al., 2006; Zhou et al., 2015).

Although most rat pancreatic beta‐cell neogenesis occurs in the prenatal period, a burst in beta‐cell growth has been observed in the rat neonate (Gregg et al., 2012). The neonatal rat beta‐cell nucleus expresses AR (Harada et al., 2019), which may be a critical regulator of islet‐cell density, beta‐cell cluster size, and consequently, neonatal rat glucose tolerance (Harada et al., 2018, 2019). Furthermore, prenatal flutamide administration has been shown to reduce male rat beta‐cell mass which resulted in glucose‐intolerance at birth and may be a result of the nuclear localization of the rat AR (Diaz‐Sanchez et al., 1995; Harada et al., 2018, 2019; Kooptiwut et al., 2015). Additionally, prenatal rat testosterone exposure may induce hyperinsulinemia and an increased HOMA‐IR, an index of insulin resistance (More, Mishra, Gopalakrishnan, et al., 2016).

We hypothesized that blockade of the androgen receptor in the neonate will alter the immediate neonatal glucose and insulin responses (and insulin sensitivity) to hypoxia in a manner that is consistent throughout neonatal life. The long‐term goal is to determine if flutamide is a consistently effective androgen receptor antagonist in the neonatal rat to utilize it to evaluate the effect of neonatal androgens on long‐term, sexually dimorphic phenotypes. We previously established the dynamics of flutamide action on the HPA‐axis in our neonatal rat model of hypoxia and suggested that flutamide may not have consistent, developmental AR antagonist activity at different ages from birth to weaning (Rolon et al., 2019). Here, we extend these studies by evaluating the effects of standard and high dose flutamide pretreatment on the neonatal insulin and glucose response to hypoxia—a common stressor in prematurity. We calculated HOMA‐IR as an index of overall insulin resistance.

## 2 METHODS

### 2.1 Animal treatment and experimental protocols

This is a follow‐up study of a cohort of neonatal rats treated with flutamide and subsequently exposed to hypoxic stress as described in detail previously (Rolon et al., 2019) and briefly summarized here. Timed‐pregnant Sprague Dawley rats (*N* = 75) were obtained from Envigo and kept in a standardized environment with food (Envigo, product #8604 which contains corn, soy, and wheat) ad libitum (Gehrand et al., 2016; Gehrand et al., 2019). Dams delivered pups spontaneously and cared for their rat pups without interruption until experimentation. Federal guidelines (https://grants.nih.gov/grants/olaw/references/phspol.htm) were followed for the use and handling of laboratory animals. Protocols were approved by the Institutional Animal Care and Use Committee of Aurora Health Care.

### 2.2 Age group of rat pups

Male and female pups were studied on four postnatal days (PD) to determine age‐dependent changes before weaning on fasting plasma glucose and insulin. Flutamide and vehicle injections were randomized to PD1, PD6, PD13, or PD20 pups that were then studied 24 hr later (at PD2, PD7, PD14, PD21) (Rolon et al., 2019).

### 2.3 Drug treatment groups

Flutamide (F9397; Sigma‐Aldrich, St. Louis, MO) or vehicle (sesame oil; S3547; Sigma‐Aldrich) pretreatment was given subcutaneously 24 hr before experimentation to allow the full effect of flutamide in the perinatal rat (Lieberburg et al., 1980). Male and female pups were randomized to standard dose flutamide (10 mg/kg), high dose flutamide (50 mg/kg) or vehicle control (10 μl/g body weight sesame oil).

### 2.4 Experiments

Pups within each drug pretreatment group were separated from their dams 24 hr after injection. Pups were randomized into baseline control, 90 min normoxia time control, and 90 min hypoxia (8% O_2_). An inspired O_2_ of 8% gives a SpO_2_ of 80%–85% which is clinically relevant in the NICU (Borenstein‐Levin et al., 2020; Bruder, Taylor, et al., 2008; Cumpstey et al., 2020). The 90‐min normoxic samples are the control for hypoxia and serve to evaluate the effects of fasting on glucose and insulin without and with concomitant hypoxia. For the purposes of this report, we use “separation” and “fasting” synonymously depending on the context. Trunk blood samples were obtained by decapitation and collected in tubes containing K_2_EDTA. In postnatal day 2 (PD2) pups, 2–3 samples were pooled and considered *n* = 1 for statistical analyses (Bruder et al., 2011). PD7, PD14, and PD21 samples consisted of one pup per K_2_EDTA tube.

### 2.5 Plasma hormone assays and statistical analyses

Plasma insulin was measured via ELISA (Crystal Chem, Downers Grove, IL) and plasma glucose was measured by spectrophotometry using the glucose oxidase method (Pointe Scientific, Canton, MI) as described previously (Guenther et al., 2012). The 90 min (separated/fasted) normoxic and hypoxia data were used to calculate the homeostatic model assessment of insulin resistance (HOMA‐IR), a validated index of insulin resistance in rats at different stages of development, as the product of fasting plasma glucose and fasting plasma insulin divided by a constant (Aref et al., 2013; Cacho et al., 2008; Wan et al., 2014; Wang et al., 2015, 2018).

Data analysis was performed by three‐way ANOVA with and without log_10_ transformation and Duncan's multiple range test (SigmaPlot 12.5; Systat Software, Inc.). In order to explore the data sets with high residual variance by three‐way ANOVA, Mann–Whitney Rank Sum Test, *t* tests, and one‐ and two‐way ANOVAs were used and annotated in each figure legend. *p* < .05 was considered statistically significant and values are reported as mean ± *SEM*.

## 3 RESULTS

For the purposes of clarity and communication, and following our previous approach (Rolon et al., 2019), we have presented the data as follows: We first describe the results from the vehicle injections compared to the standard dose of flutamide (10 mg/kg) used in our previous study chosen because it does not have long‐term feminizing effects (Gray et al., 2006; McCormick & Mahoney, 1999). The second section of the Results presents the data from the standard dose of flutamide compared to the higher dose (50 mg/kg) as described in the Methods section. Throughout the results section, the abbreviation PD stands for postnatal day.

### 3.1 Vehicle injection versus standard dose flutamide

#### 3.1.1 Baseline (control)

**Figure 1:**
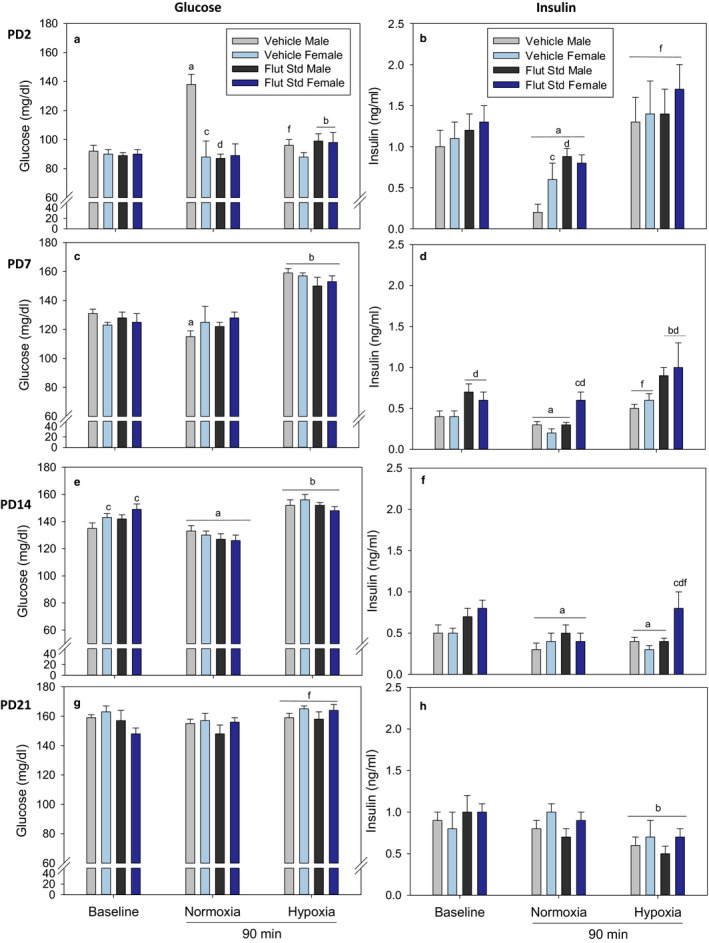
Plasma Glucose and Insulin responses to baseline, normoxia, and hypoxia in postnatal day 2 (PD2), PD7, PD14, PD21 pups after pretreatment with vehicle or standard dose flutamide (10 mg/kg). Values are mean ± *SE*. *N* values are PD2 Vehicle/Male (8–10), Vehicle/Female (10–11), Flutamide/Male (10–12), Flutamide/Female (11–14); PD7 Vehicle/Male (9–11), Vehicle/Female (8–9), Flutamide/Male (10–12), Flutamide/Female (10–12); PD14 Vehicle/Male (9–12), Vehicle/Female (10–11), Flutamide/Male (13), Flutamide/Female (10–13); PD21 Vehicle/Male (8–9), Vehicle/Female (8–9), Flutamide/Male (7–9), Flutamide/Female (10). ^a^Different from baseline; ^b^Different from baseline and normoxia; ^c^Different within sex; ^d^Effect of flutamide within age; ^f^Hypoxia different from normoxia. For glucose panels (a, c, e, g): All statistics by three‐way ANOVA. For insulin panels (b, d, f, h): Statistics by three‐way ANOVA except: one‐way ANOVA: ^d^ in panel b Normoxia, ^a,c,d^ in panel d Normoxia; ^c,d^ in panel f Hypoxia. Two‐way ANOVA: ^f^ in panel f Hypoxia

Baseline plasma glucose concentrations increased between PD2 and PD7 and increased between PD14 and PD21 in male and female pups (panels a⇾c and e⇾g) (Figure 1). There was almost a twofold difference in plasma glucose concentrations in PD21 pups compared to PD2 pups. Plasma glucose concentrations were significantly greater in PD14 female pups compared to males. Baseline plasma insulin concentrations were highest in PD2 male and female pups. Furthermore, baseline plasma insulin concentrations decreased from PD2 (panel b) to PD7 (panel d) in male and female pups, and this change was not sexually dimorphic. Finally, baseline plasma insulin concentrations were lowest in PD7 and PD14 and tended to increase from there in PD21 pups.

#### 3.1.2 Effect of standard dose flutamide in baseline (control)

There was no effect of flutamide on plasma glucose concentrations across all ages in male and female pups within the baseline. Flutamide pretreatment increased plasma insulin in PD7 male and female pups.

#### 3.1.3 Effect of standard dose flutamide in normoxia

Plasma glucose concentrations were significantly increased in PD2 vehicle‐treated male pups at 90 min of normoxia (the fasting time control for hypoxia) compared to baseline; this was blocked by the standard dose of flutamide. Plasma glucose concentrations were significantly decreased in PD7 males compared to baseline. However, there was no effect of standard dose flutamide on plasma glucose in PD7 male and female pups. Overall, normoxia (fasting) lowered plasma glucose in PD14 male and female pups compared to baseline, irrespective of treatment with standard dose flutamide. There was no effect of treatment or standard dose flutamide on plasma glucose concentrations in PD21 male and female pups.

Plasma insulin was decreased in normoxic PD2, PD7, and PD14 male and female pups treated with vehicle (fasting alone). In PD2 pups, the decrease was greater in males that were in concordance with the larger increase in plasma glucose (Figure 1a). Standard dose flutamide attenuated the decrease in plasma insulin in PD2 males with normoxia (fasting), again paralleling the attenuation of the increased glucose. Standard dose flutamide increased plasma insulin in PD7 female pups with normoxia, but this drug effect was not observed in male pups. There was no effect of standard dose flutamide in PD14 male and female pups with normoxia (fasting). With 90 min of normoxia or hypoxia, there was no effect of standard dose flutamide on plasma insulin concentrations in PD21 male and female pups.

#### 3.1.4 Effect of standard dose flutamide with hypoxia

Standard dose flutamide increased plasma glucose concentrations in PD2 male and female pups exposed to hypoxia compared to baseline and normoxia. Although hypoxia increased plasma glucose concentrations in PD7 and PD14 male and female pups compared to baseline and normoxia, there was no effect of standard dose flutamide on plasma glucose. While hypoxia increased plasma glucose concentrations in PD21 male and female pups compared to normoxia, there was no effect of standard dose flutamide on plasma glucose.

Plasma insulin concentrations were increased in PD2 male and female pups with hypoxia compared to normoxia, but there was no effect of standard dose flutamide on plasma insulin. Plasma insulin concentrations were increased in PD7 male and female pups with hypoxia compared to normoxia. Standard dose flutamide further amplified the increase in plasma insulin concentrations in PD7 hypoxic male and female pups. Plasma insulin was decreased in PD14 male and female pups with hypoxia compared to baseline. Flutamide attenuated the decrease in plasma insulin in PD14 hypoxic female pups, but not in male pups. Plasma insulin was decreased in PD21 male and female pups with hypoxia compared to baseline and normoxia. There was no effect of standard dose flutamide on plasma insulin concentrations in PD21 male and female pups.

#### 3.1.5 Integration of vehicle injection versus standard dose flutamide findings

In most ages, injection of the standard dose of flutamide had no major effect on plasma glucose concentrations across all ages (Figure 1). PD2 normoxic vehicle‐treated male pups had elevated plasma glucose that was not observed in the other age groups. Hypoxia tended to increase plasma glucose in most age groups. This change was not present in the female PD2 pups that received the vehicle. The major effect of the standard dose of flutamide on plasma insulin concentration was an increase in PD7 and PD14 female pups under hypoxia. There was no major effect of standard dose flutamide on plasma insulin concentrations in PD2 and PD21. Normoxia (fasting) tended to lower plasma insulin at PD2, PD7, and PD14. Hypoxia tended to lower plasma insulin at PD14 and PD21. PD7 and PD14 female pups, however, tended to maintain elevated plasma insulin levels under normoxia and hypoxia compared to male pups.

### 3.2 Standard dose versus high dose flutamide

**Figure 2:**
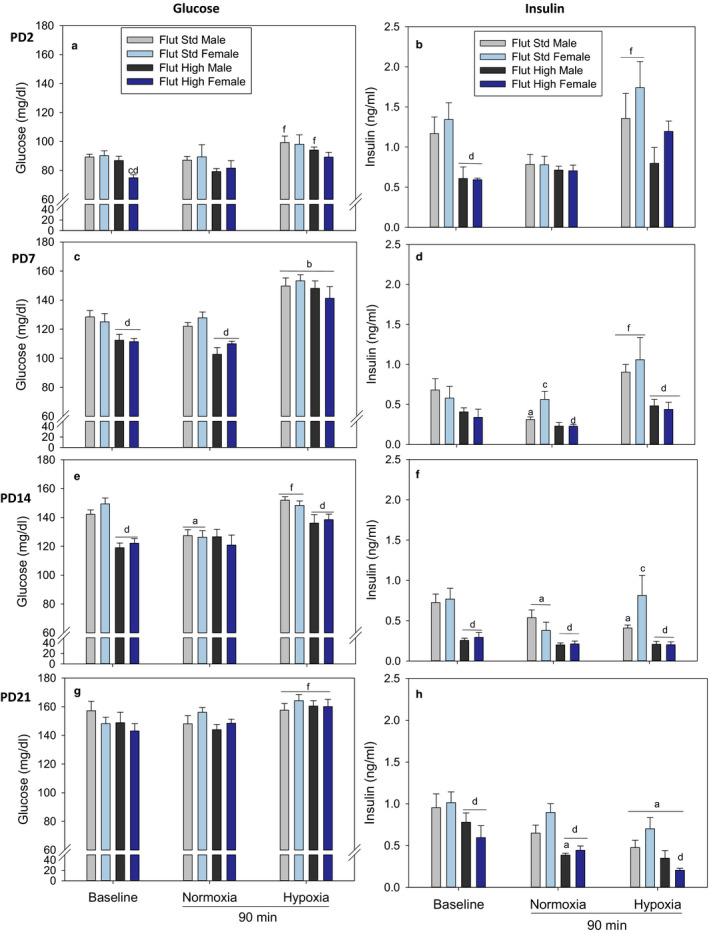
Plasma Glucose and Insulin responses to baseline, normoxia, and hypoxia in postnatal day 2 (PD2), PD7, PD14, PD21 pups after pretreatment with standard dose flutamide (10 mg/kg) or high dose flutamide (50 mg/kg). Values are mean ± *SE*. *N* values are PD2 Flutamide/Male (10–12), Flutamide/Female (11–14), High Dose (HD) Flutamide (2–4); PD7 Flutamide/Male (10–12), Flutamide/Female (10–12), HD (3–8); PD14 Flutamide/Male (13), Flutamide/Female (10–13), HD (8); PD21 Flutamide/Male (7–9), Flutamide/Female (10), HD (4–6). ^a^Different from baseline; ^b^Different from baseline and normoxia; ^c^Different within sex; ^d^Effect of flutamide within age; ^f^Hypoxia different from normoxia. For glucose panels (a, c, e, g): All statistics by three‐way ANOVA except: *t* test: ^c,f^ panel a; one‐way ANOVA; ^d,f^ in panel e. For all insulin panels (b, d, f, h): Statistics by three‐way ANOVA except: one‐way ANOVA: ^c,d^ in panel d Normoxia; ^d^ in panel f, ^d^ in panel h Hypoxia. *t* test: ^d^ panel h Baseline and Normoxia

As explained at the beginning of the Results section and in keeping with our previous approach to presenting this type of data with clarity, we have replotted the standard dose of flutamide described above for comparison with the high dose of flutamide treatments (Figure 2). This description will focus on the circumstances where high dose flutamide led to a different response than with standard dose flutamide.

#### 3.2.1 Effect of high dose flutamide in baseline control

High dose flutamide decreased plasma glucose concentrations in PD2 female (but not male) pups within baseline compared to standard dose flutamide. High dose flutamide decreased plasma glucose concentrations in PD7 and PD14 male and female pups compared to the standard dose. There was no difference in the effect of high dose flutamide on plasma glucose in PD21 male and female pups within baseline compared to the standard dose.

High dose flutamide decreased plasma insulin in PD2, PD14, and PD21 male and female pups within baseline compared to the standard dose. There was no difference of high dose flutamide on plasma insulin in PD7 male and female pups with baseline compared to the standard dose.

#### 3.2.2 Effect of high dose flutamide in normoxia

There was no difference in the effect of high dose flutamide on plasma glucose concentrations in PD2, PD14 and PD21 male and female pups with normoxia (fasting) compared to standard dose flutamide. High dose flutamide decreased plasma glucose in PD7 male and female pups with normoxia compared to the standard dose.

There was no difference in the effect of high dose flutamide on plasma insulin concentrations in PD2 male and female pups with normoxia compared to the standard dose. High dose flutamide decreased plasma insulin in PD7 females (but not in males) as well as in PD14 and PD21 male and female pups with normoxia compared to the standard dose.

#### 3.2.3 Effect of high dose flutamide in hypoxia

There was no difference in the effect of high dose flutamide on plasma glucose concentrations in PD2, PD7, and PD21 male and female pups with hypoxia compared to the standard dose. High dose flutamide decreased plasma glucose in PD14 male and female pups with hypoxia compared to the standard dose.

There was no difference in the effect of high dose flutamide on plasma insulin concentrations in PD2 male and female pups with hypoxia compared to standard dose flutamide. High dose flutamide decreased plasma insulin in PD7 and PD14 male and female pups as well as in PD21 female (but not male) pups.

#### 3.2.4 Integration of standard dose versus high dose flutamide findings

High dose flutamide decreased plasma glucose compared to standard treatment in all age groups with the exception of PD21 hypoxic male pups (Figure 2). The effect of high dose flutamide on plasma glucose was most prominent in PD7 and PD14 pups. High dose flutamide consistently lowered plasma insulin across all age groups and in both sexes compared to standard treatment. While the magnitude of the change in plasma insulin varied as a result of post‐natal age, the relative effect of high dose flutamide on plasma insulin was consistent.

### 3.3 HOMA‐IR

**Figure 3:**
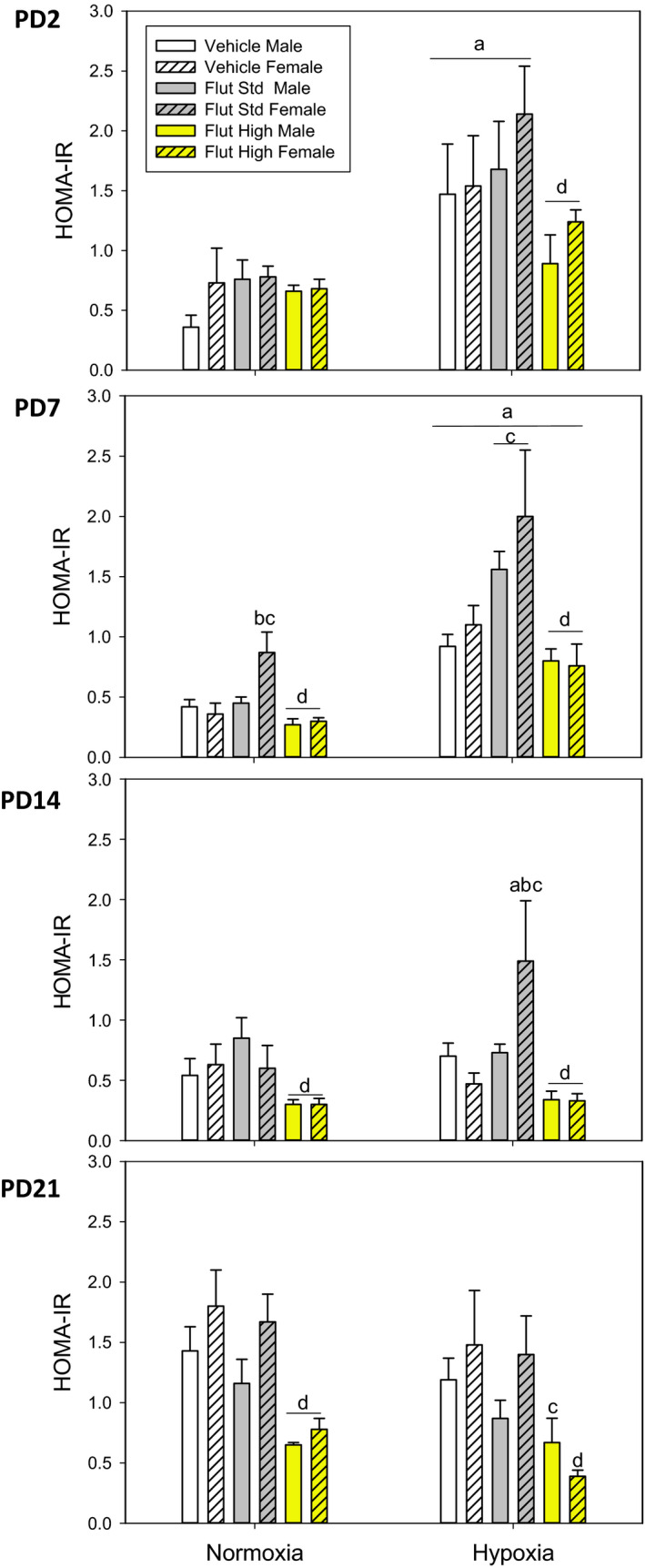
HOMA‐IR responses to normoxia and hypoxia in postnatal day 2 (PD2), PD7, PD14, PD21 pups after pretreatment with standard dose flutamide (10 mg/kg) or high dose flutamide (50 mg/kg). Values are mean ± *SE*. *N* values are PD2 Vehicle/Male (8–10), Vehicle/Female (10–11), PD2 Flutamide/Male (10–12), Flutamide/Female (11–14), High Dose (HD) Flutamide (2–4); PD7 Vehicle/Male (9–11), Vehicle/Female (8–9), PD7 Flutamide/Male (10–12), Flutamide/Female (10–12), HD(3–8); PD14 Vehicle/Male (9–12), Vehicle/Female (10–11), PD14 Flutamide/Male (13), Flutamide/Female (10–13), HD (8); PD21 Vehicle/Male (8–9), Vehicle/Female (8–9), PD21 Flutamide/Male (7–9), Flutamide/Female (10), HD (4–6). ^a^Different from normoxia; ^b^Different within sex; ^c^Different from the vehicle; ^d^High dose of flutamide different from the standard dose of flutamide. All statistics by three‐way ANOVA except: two‐way ANOVA: ^d^ in PD2, ^b,c^ in PD7, ^a,b,c^ in PD14, ^c^ in PD21

Figure 3 shows the HOMA‐IR, an index of insulin resistance calculated from fasting plasma glucose and insulin, in the PD2, PD7, PD14, and PD21 rat pups after 90 min of maternal separation (fasting) under normoxic (control) versus hypoxic conditions. Overall, the younger pups (PD2 and PD7) receiving vehicle or the standard dose of flutamide showed an increase in HOMA‐IR (insulin resistance) at 90 min of hypoxia. Except for the PD2 normoxic female pups, the higher dose of flutamide decreased HOMA‐IR (lessened insulin resistance). There were very few male‐female differences in any of the HOMA‐IR data except for the normoxic female pups at PD7 and the hypoxic female pups at PD14 with the standard dose of flutamide. Although not statistically significant, there was a tendency for female normoxic and hypoxic PD21 pups with vehicle or standard flutamide to have higher HOMA‐IR.

## 4 DISCUSSION

This study was motivated by our previous studies showing that neonatal stressors alter the adult control of the HPA axis and metabolic biomarkers including insulin, glucose, and HOMA‐IR in a sexually dimorphic manner (Gehrand et al., 2016, 2018; Raff et al., 2018). We thought it was possible that this long‐term programing could be a result of the stress‐induced alterations in testosterone in the neonatal male rat. Flutamide has been used as an agent for androgen receptor modulation in the neonatal rat (Gray et al., 2006; McCormick & Mahoney, 1999). We have previously used flutamide in our neonatal rat model that involves separation (fasting) and hypoxia and mimics human prematurity and clinical intensive care (Rolon et al., 2019). We have now continued our previous study using flutamide in this model to evaluate plasma glucose and insulin dynamics.

Maternal‐neonatal separation (fasting) and hypoxia are potent, additive neonatal stressors leading to an increase in corticosterone and, therefore, apparent insulin resistance (Dallman et al., 1995; Morakinyo et al., 2019; Strack et al., 1995). If so and based on previous findings by others showing that testosterone increases insulin secretion (More, Mishra, Hankins, et al., 2016; Morimoto et al., 2001, 2005; Ramaswamy et al., 2016), we thought it possible that flutamide, an AR antagonist, would attenuate the plasma insulin in response to separation and hypoxia and possibly improve insulin sensitivity. Furthermore, we might expect reciprocal changes in plasma glucose due to the flutamide‐mediated modulation of pancreatic islet cell activity (Harada et al., 2019; Kupreeva et al., 2019). Or, it could be possible that corticosterone‐driven insulin resistance (Dallman et al., 1995; Jimeno et al., 2018; Morakinyo et al., 2019; Strack et al., 1995; Tadaishi et al., 2018) could increase plasma glucose which would then stimulate the release in insulin, and that this could be modulated by AR blockade.

The major, generalized findings of this study were that (a) during normoxic and hypoxic separation (fasting), flutamide increased insulin secretion in postnatal day 2 (PD2), PD7, and PD14 pups in the absence of major changes in plasma glucose, (b) normoxic separation (fasting) with vehicle treatment decreased plasma glucose and insulin in PD2 PD7, and PD14 female pups, (c) normoxic separation (90 min of fasting) increased plasma glucose and decreased plasma insulin in PD2 males, (d) hypoxic separation increased plasma glucose across all ages without significant sexual dimorphism, (e) the PD21 pup was able to maintain higher levels of fasting plasma glucose compared to younger pups, (f) high dose flutamide attenuated insulin secretion compared to the standard dose of flutamide, and (g) overall, hypoxia increased HOMA‐IR (worsened insulin resistance) and the higher dose of flutamide decreased HOMA‐IR (attenuated insulin resistance).

An effect of flutamide on plasma glucose was only observed in the PD2 male. Flutamide attenuated the increase in plasma glucose in PD2 male pups with normoxic separation (90 min of fasting). This is most likely due to a flutamide‐induced increase in plasma insulin secretion in the PD2 normoxic male. There was no flutamide effect on plasma glucose in the PD2 female pup, which is probably due to lower levels of circulating plasma androgen in neonatal female rats (McCormick et al., 1998). The major results with the standard dose of flutamide (Figure 1) suggest that overall there was no effect on plasma glucose in PD7, PD14, and PD21 male and female pups. However, there were significant effects of flutamide on insulin secretion that occurred in the absence of changes in plasma glucose, as described below.

Previous studies have shown that the activity of the androgen receptor can regulate insulin secretion from islet beta‐cells in rats and mice (Harada et al., 2018, 2019; Kooptiwut et al., 2015; Li et al., 2008; Xu et al., 2017). There seems to be an overall protective effect in rat islet cells exposed to testosterone via attenuation of superoxide production and decreased Caspase 3 cleavage under a high‐glucose environment (Kooptiwut et al., 2015). Furthermore, androgens may amplify GLP‐1 mediated insulin secretion via a cAMP‐dependent protein kinase pathway in mice islet cells (Xu et al., 2017). Interestingly, our study demonstrated that standard dose flutamide, an androgen receptor blocker, paradoxically augmented insulin secretion in the hypoxic rat. This observed drug effect can be explained by the indirect effects of flutamide as described below. Exposure to hypoxia increases plasma corticosterone in the neonatal rat (Bruder, Taylor, et al., 2008; Gehrand et al., 2019). Corticosterone, the rodent endogenous glucocorticoid, functions to increase plasma glucose in the hypoxic rat via gluconeogenesis and glycogenolysis as well as increased insulin resistance (Jimeno et al., 2018; Tadaishi et al., 2018). One could expect, therefore, a concomitant increase in plasma insulin via classic glucose‐mediated effects on the pancreatic islet beta‐cell (Morakinyo et al., 2019). We have previously demonstrated that flutamide augments the corticosterone response to hypoxia in the neonatal rat (Rolon et al., 2019) which may have indirectly increased insulin secretion. It is possible that standard dose flutamide did not achieve sufficient concentrations in the rat pancreas and so the *direct* effects of beta‐cell androgen receptor blockade (i.e., plasma insulin attenuation) were not observed. In fact, high dose flutamide led to decreased insulin secretion, most likely due to sufficient androgen receptor blockade in the rat islet beta‐cell and/or improvement in the sensitivity to insulin as discussed below (Harada et al., 2019; Kupreeva et al., 2019).

As stated above, we have previously demonstrated that flutamide augments the corticosterone response to hypoxia which may have led to an *indirect* increase in plasma insulin in the hypoxic PD7 and PD14 rat. This was not observed in the PD21 hypoxic pup. We have previously shown that the neonatal pup probably achieves the adult level of adrenocortical steroidogenic response to hypoxia by PD21, which is just before weaning (Rolon et al., 2019; Wood & Walker, 2015). In the PD21 pup, a maximum corticosterone response may have already led to a maximum secretion of insulin, such that treatment with flutamide resulted in no effect on plasma insulin.

We used HOMA‐IR as an index of insulin resistance and found, not surprisingly that it was increased during hypoxia in PD2 and PD7 pups. This is likely due, at least in part, to a stress‐induced increase in counterregulatory hormones such as corticosterone (Dallman et al., 1995; Morakinyo et al., 2019; Strack et al., 1995). However, hypoxia did not increase HOMA‐IR in PD21 pups in whom we have shown very large increases in corticosterone (Rolon et al., 2019). One possibility is that HOMA‐IR is not a good index of insulin resistance in very young neonates. Another is that the older pups have a better developed ability to maintain insulin sensitivity despite increases in corticosterone (Aref et al., 2013). The other interesting finding is the decrease in HOMA‐IR with high dose of flutamide. This is consistent with the known detrimental effects of androgen on insulin sensitivity as well as the possibility that flutamide is a useful therapy to improve insulin sensitivity (Harada et al., 2018, 2019; Li et al., 2008; Pignatelli et al., 2006; Zhou et al., 2015).

A limitation of the neonatal rat model we use is that we separate the pups from their lactating dams (so the dams are not exposed to hypoxia) which means that separation involves the lack of contact with the other nurturing aspects of maternal care independent of the fact that the pups are not suckling during 90 min. Exposing the dams to hypoxia does have effects on their milk production and composition (Bruder, Van Hoof, et al., 2008). For the current study, fasting was emphasized because it is optimal to evaluate changes in HOMA‐IR. Therefore, there is no “separated/fed” control group. That said, as we have stated, it is considered normal behavior for lactating dams to leave the pups for periods of 60–90 min so the fasting effect should be less important in the long term than being in a cage without the presence of the dam (Calhoun, 1962).

We conclude that the effects of standard dose flutamide on plasma insulin and glucose occur in a differential and age‐dependent manner during the first 3 weeks of neonatal life, which renders standard doses of flutamide a less than the optimal agent for studying the effects of androgen on neonatal pancreatic endocrine function and common neonatal pathologies such as maternal hyperandrogenism, glucose intolerance, and insulin resistance. However, the higher dose of flutamide in the neonate was consistently and clearly effective, suggesting that it is a better choice for these types of neonatal studies.

We elucidated a potentially unique role of androgen‐receptor mediated control of neonatal endocrine function, possibly due to a direct effect of neonatal testosterone secretion in conjunction with simultaneous and indirect regulatory effects of the HPA‐axis or other counterregulatory systems on pancreatic islet cells. For example, we suggest that flutamide may indirectly increase plasma insulin primarily via augmentation of the adrenocortical steroidogenic response to hypoxia shown in our previous study (Rolon et al., 2019). Furthermore, flutamide may have direct effects on gluconeogenesis in a sex‐dependent manner, which may have permanent effects on pancreatic function and islet cell mass observed into adulthood (Harada et al., 2019). While most beta‐cell neogenesis occurs before birth, beta to alpha‐cell ratios double neonatally (Gregg et al., 2012) and this may be regulated by gonadal androgen secretion (Harada et al., 2018, 2019). The dose of flutamide chosen seems to be critical. The short and long‐term effects of the HPA‐axis and gonadal androgen secretion on adult gene expression and endocrine phenotype continues to be an important area of study, especially in regard to sex differences in the development of disease affecting pancreatic cell function.

## CONFLICT OF INTEREST

The authors have no conflicts of interest.

## AUTHOR CONTRIBUTION

S.R., A.G., and H.R. conceived the idea and the experimental design. All authors performed biostatistical analyses, constructed the figures, and performed the hormone assays and calculations. S.R., C.H., M.G., M.G., J.P., and A.G. performed animal experimentation. S.R. wrote the first draft of the manuscript. S.R., A.G., J.P., and H.R. performed the first revisions of the manuscript. All authors reviewed and helped revise the penultimate draft of the manuscript and approved the final submission.

## Data Availability

All the data supporting the published results are stored on the Advocate Aurora Health Care network and backed‐up on a USB flash drive and CD‐ROM.
